# Antifungal, Insecticidal, and Repellent Activities of *Rosmarinus officinalis* Essential Oil and Molecular Docking of Its Constituents against Acetylcholinesterase and *β*-Tubulin

**DOI:** 10.1155/2024/5558041

**Published:** 2024-08-05

**Authors:** Ghizlane Houzi, Youness El abdali, Ghada Beniaich, Mohamed Chebaibi, Mohamed Taibi, Amine Elbouzidi, Samiha Kaioua, Abdeslam Asehraou, Mohamed Addi, Khalid Chaabane, Rachid Flouchi, Aimad Allali, Soad Khal-Layoun

**Affiliations:** ^1^ Laboratory of Biology and Health Faculty of Sciences University of Ibn Tofail, Kenitra, Morocco; ^2^ Laboratory of Biotechnology, Environment, Agri-Food and Health Faculty of Sciences Dhar El Mahraz Sidi Mohammed Ben Abdellah University, Fez 30000, Morocco; ^3^ Laboratory of Engineering, Electrochemistry, Modeling and Environment (LIEME) Faculty of Sciences Dhar El Mahraz Sidi Mohamed Ben Abdellah University, Fez, Morocco; ^4^ Higher Institute of Nursing Professions and Health Techniques, Fez 30000, Morocco; ^5^ Biomedical and Translational Research Laboratory Faculty of Medicine and Pharmacy of Fez Sidi Mohamed Ben Abdellah University, Fez 30000, Morocco; ^6^ Laboratoire d'Amélioration des Productions Agricoles, Biotechnologie et Environnement (LAPABE) Faculté des Sciences Université Mohammed Premier, Oujda 60000, Morocco; ^7^ Laboratory of Plant, Animal and Agro-Industry Productions Faculty of Sciences University of Ibn Tofail, Kenitra, Morocco; ^8^ Laboratory of Bioresources, Biotechnology, Ethnopharmacology and Health Faculty of Sciences Mohammed First University, Boulevard Mohamed VI, B.P. 717, Oujda 60000, Morocco; ^9^ Microbial Biotechnology and Bioactive Molecules Laboratory Sciences and Technologies Faculty Sidi Mohamed Ben Abdellah University, Fez, Morocco; ^10^ Ministry of Health and Social Protection High Institute of Nursing Professions and Health Techniques Annex Taza, Fez, Morocco

## Abstract

The aim of this study was to determine the phytochemical composition and evaluate the antifungal and insecticidal properties of *Rosmarinus officinalis* essential oil (EO). GC-MS was employed to analyze the phytochemical profile of the EO. The antifungal activity of the EO was assessed by calculating growth inhibition rates for *Alternaria alternata*, *Fusarium oxysporum*, and *Botrytis cinerea*. Repellent capacity and toxicity were evaluated through inhalation and contact tests on *Callosobruchus maculatus*. Molecular docking techniques were utilized to test the insecticidal and antifungal activities of rosemary EO. The analysis revealed a total of sixteen components in *R*. *officinalis* EO, with 1,8-cineole (40.80%) being the major constituent, followed by *α*-pinene (26.18%) and camphor (19.53%). Antifungal evaluation demonstrated a significant inhibitory impact on the mycelial growth of the tested fungi, with complete inhibition observed against *B. cinerea*. In terms of insecticidal capacity, the EO induced complete mortality of *C. maculatus* adults at a concentration of 1 *μ*L/L air, with an inhalation test LC_50_ value of 0.62 *μ*L/L air. Concentration-dependent reductions were observed in the number of both laid eggs and emerged insects, reaching a 99.36% reduction. The EO also exhibited a moderate effectiveness in repelling insects, with an average repellency rate of 50.83%. *In silico* analysis identified borneol as the most active molecule against insect acetylcholinesterase (PDB: 6ARY) with a Glide score of −7.254 kcal/mol. *α*-Caryophyllene showed the highest activity against *B. cinerea β*-tubulin (PDB: 3N2G) with a Glide score of −7.025 kcal/mol. These findings suggest that the EO derived from Moroccan *Rosmarinus officinalis* has potential as an effective natural agent against pathogenic fungi and could serve as a sustainable and environmentally friendly alternative as a bioinsecticide.

## 1. Introduction

Chickpea (*Cicer arietinum*), globally renowned for its nutritional richness in dietary protein, minerals, and vitamins, faces considerable challenges in storage due to yield losses inflicted by various pests, notably bruchids. Among these, *Callosobruchus maculatus*, commonly known as the chickpea weevil, emerges as a particularly destructive insect. This species has the ability to lay eggs in cultivated fields and storage warehouses. The larvae, feeding internally on the seeds, present formidable challenges for effective management using chemical insecticides [1]. The loss of yield in leguminous crops during storage, primarily caused by various insects, particularly bruchids, poses a significant challenge for traders and farmers [2]. *C*. *maculatus*, known as the chickpea weevil, stands out as one of the most destructive pests affecting chickpeas. This insect exhibits the ability to deposit eggs in both cultivated fields and storage sites, and its larvae, which internally consume the seeds, prove challenging to manage using chemical insecticides [1]. Synthetic insecticides are frequently used to control pests in agricultural crops. Consequently, their excessive and widespread use has resulted in adverse impacts on human health, environmental contamination, pesticide residues on fruits, vegetables, and seeds, as well as the development of pest resistance and weed resistance. At present, biopesticides derived from essential oils (EOs) are gaining prominence as a promising alternative to alleviate the negative impacts of synthetic pesticides. These biopesticides are known for their specificity in targeting agricultural pests and their biodegradable nature [3].

Filamentous fungi represent the most widespread group of pathogenic microorganisms affecting crops, leading to a decline in agricultural productivity, deterioration in quality, and substantial economic losses [4]. Among these, mycotoxigenic fungi are renowned for their capability to infest various cereals, vegetables, spices, and fruits. These molds generate various mycotoxins known for their genotoxic, carcinogenic, and immunosuppressive effects on human health [5]. Currently, the main methods for preventing fungal infections involve the adoption of resistant cultivars and phytosanitary treatments [6]. However, to limit the accumulation of residues in the soil and combat the constant evolution of new fungal strains resistant to chemical treatments, it is imperative to adopt new, environmentally friendly approaches to crop protection [7]. In this situation, EOs are emerging as potential substitutes for synthetic fungicides, thereby enhancing the means of protection against these plant pathogens [8].

Recently, EOs derived from aromatic and medicinal plants have garnered significant interest due to their diverse biological effects [9]. *Rosmarinus officinalis* (Lamiaceae), native to the Mediterranean basin, possesses an essential oil that contributes to its remarkable properties. This oil is acknowledged for its antioxidant, antidiabetic, anti-inflammatory, anticarcinogenic, antimicrobial, hepatoprotective, antinociceptive, antiulcerogenic, antithrombotic, and diuretic effects [10]. Furthermore, rosemary essential oil comprises several monoterpenes like limonene and 1,8-cineole, which have exhibited antifungal properties and have demonstrated success in the control of insects [8, 11, 12]. Despite this, there remains a paucity of data and research regarding the bioactivities of Moroccan chemotypes of *R. officinalis* EO, particularly concerning phytopathogenic fungi and agricultural pests. Therefore, the purpose of the present study was to characterize and assess the antifungal activity of the essential oil extracted from the aerial parts of *R. officinalis* against specific phytopathogenic fungi responsible for leguminous contamination. Moreover, this study explores the insecticidal and repellent capacities of the EO derived from this emblematic plant of the Moroccan pharmacopoeia against *Callosobruchus maculatus*, a notorious pest affecting chickpea seeds. Additionally, to understand the mechanism of action of *Rosmarinus officinalis* EO during insecticidal and antifungal activities, molecular docking studies, *in silico*, against insect acetylcholinesterase and *Botrytis cinereaβ*-tubulin were conducted.

## 2. Materials & Methods

### 2.1. Plant Material

In this study, the plant material used consists of *Rosmarinus officinalis* leaves, harvested in the Boulemane (Skoura) region of Morocco in May 2021. Laboratory botanists identified the plant samples by referring to various botanical works and plant catalogs. Subsequently, the samples were subjected to a cleaning process and left to dry in the shade and open air for 15 days before the extraction process began.

### 2.2. Essential Oil Extraction

A quantity of 200 grams of dried *R. officinalis* leaves was subjected to hydrodistillation for 3 hours using a Clevenger-type apparatus with 1000 mL of distilled water, following the standard procedure outlined in the European Pharmacopoeia [13]. The resulting oils were subsequently dried using anhydrous sodium sulfate and then stored in dark conditions at 4–5°C until testing and analysis. The EO yield, calculated relative to the weight of the dried plant material, was expressed as a percentage (*v/w*) [14].

### 2.3. Chemical Analysis of the Essential Oil

The present investigation employed GC-MS to meticulously discern the diverse phytochemical constituents inherent in the EO derived from *R*. *officinalis*. The analytical protocol entailed the utilization of a GC Agilent Technologies 6890 N Network gas-phase chromatograph, featuring an HP-5MS capillary column (30.0 m × 0.250 mm × 0.250 *μ*m film thickness), strategically situated in Little Falls, CA, USA. The flame ionization detector (FID) employed in this study was judiciously set at a precise temperature of 250.0°C. The injection of a 1.00 *μ*L volume was executed with meticulous care, adopting a split mode at the designated temperature of 250.0°C. Helium served as the carrier gas, maintaining a steadfast flow rate of 1.00 mL/min throughout the chromatographic separation process. The temperature profile of the chromatographic column was systematically programmed, advancing at a controlled rate of 0.5°C/min, spanning from an initial temperature of 35°C to a final temperature of 250.0°C. This deliberate temperature programming strategy facilitated an in-depth exploration of the volatile compounds within the EO, ensuring a nuanced separation and analytical resolution. To elucidate and identify the constituents within the essential oil, a comprehensive strategy was undertaken. Kovats retention indices (RIs) were meticulously applied, cross-referencing against a systematically constructed homologous series of n-alkanes. Additionally, NIST MS Library (v. 2.0) was judiciously employed as the mass spectral database to enhance the precision of compound identification. Through the integration of these sophisticated analytical methodologies, the investigation achieved a comprehensive and precise characterization of the phytochemical profile within *R*. *officinalis* EO [15, 16].

### 2.4. Antifungal Activity of Essential Oil

#### 2.4.1. Culture Conditions and Strains Used

In the context of this research endeavor, three strains of filamentous fungi, namely, *Botrytis cinerea*, *Fusarium oxysporum*, and *Alternaria alternata*, were purposefully selected for scrutiny. These specific fungal strains have garnered recognition for their dual attributes of mycotoxigenicity and phytopathogenicity, thus assuming pivotal roles in the attenuation of agricultural yields and the compromise of overall production quality [8]. It is imperative to note that the inclusion of these fungal strains in the study was facilitated through collaboration with the esteemed Hassan II Institute of Agronomy and Veterinary Sciences situated in Rabat (Morocco).

The meticulous preparation of spore suspensions involved the cultivation of 7-day-old cultures of the fungi on potato dextrose agar (PDA) medium within tubes infused with a 0.9% NaCl solution. Following this cultivation period, the quantification of spore counts was methodically executed using a Malassez cell, and the resultant suspensions underwent judicious dilution to attain an inoculum concentration of approximately 10^6^ spores/mL [17]. This rigorous methodology not only ensured the precision of spore suspension preparation but also laid the foundation for subsequent phases of the study, thereby enhancing the scientific rigor and reliability of the experimental procedures.

#### 2.4.2. Disk Diffusion Method

The examination of the antifungal properties intrinsic to *Rosmarinus officinalis* EO was systematically conducted employing the disk diffusion assay, a technique well-established in prior scientific endeavors [3]. In the initial phase, Petri plates with a standardized diameter of 90 mm were meticulously prepared, housing a uniform distribution of the Czapek-Dox agar medium. The agar medium underwent central inoculation with a precisely measured 100 *μ*L spot containing an inoculum of established concentration at 10^6^ spores/mL. After the inoculation, sterile Whatman filter paper disks, measuring 5 mm in diameter, were thoroughly impregnated with *R*. *officinalis* EO at 5, 10, and 40 *μ*g/mL concentrations, utilizing dimethyl sulfoxide (DMSO) as the solvent. These EO-laden disks were judiciously positioned on the surface of the agar medium. Additionally, control Petri plates, inoculated with 10 *μ*L per disk of DMSO, were included to function as negative controls for fungal growth. To uphold the experimental integrity, Petri plates were hermetically sealed with parafilm and subjected to a carefully regulated incubation period of 6 days at 25°C [8]. Noteworthy is the implementation of three replicates for each concentration of the EO, a critical practice to ensure the statistical robustness and reliability of the empirical findings. Throughout the incubation period, meticulous daily observations were recorded, capturing precise measurements of mycelial growth. The quantification of the inhibitory effect on fungal growth, relative to the control, was accomplished through the application of the following formula:

(1) $$\begin{array}{c} \text{mycelial growth inhibition }\left(\%\right)=\left[\frac{\left(\text{dc }-\text{ dt}\right)}{\text{dc}}\right]\times 100. \end{array}$$

In this formulation, dc and dt represent the average diameter (in millimeters) of fungal mycelial growth in the control and treated fungal strains, respectively. This comprehensive and systematic approach not only facilitated a nuanced exploration of the antifungal efficacy of *Rosmarinus officinalis* EO but also ensured the methodological rigor and replicability of the experimental findings within an academic context.

### 2.5. Insecticidal Activity of Essential Oil

#### 2.5.1. Insect Collection and Breeding Conditions

The investigation into insecticidal activity intricately focused on the examination of *Callosobruchus maculatus*, a significant pest exerting its impact on chickpeas. The cultivation of bruchids, the larvae of this insect, was purposefully undertaken within glass jars, utilizing chickpea (*Cicer arietinum*) seeds as the substrate. A paramount consideration in this study was the meticulous maintenance of controlled environmental conditions to ensure the reliability and consistency of the experimental outcomes. These glass jars served as microcosms for the cultivation of the insect population, sustaining a carefully regulated temperature of 25°C. The relative humidity levels were maintained at 65 ± 5%, and a standardized photoperiod of 14 hours of illumination followed by 10 hours of darkness was rigorously imposed. This level of environmental control persisted across multiple generations of the insect population, imparting a level of stability crucial for the systematic study of their behavior and susceptibility to insecticidal agents [15]. Such precision in environmental control not only mirrors the natural habitat of the insect but also establishes a robust foundation for the comprehensive assessment of insecticidal efficacy against *Callosobruchus maculatus.*

#### 2.5.2. Essential Oil's Toxicity against *C. maculatus*

*(1) Toxicity of the Essential Oil by Contact Test*. The quantification of essential oil concentrations, expressed in microliters per liter (*μ*L/L) relative to the enclosed air volume in the experimental jars, has been rigorously established. Each Petri plate, serving as a controlled experimental unit, was meticulously equipped with 100 grams of *Cicer arietinum* seeds and featured a filter paper disk infused with distinct concentrations (1, 5, 10, and 20 *μ*L/L) of *R*. *officinalis* EO. To ensure a representative insect sample, ten adult *C*. *maculatus* insects comprising five males and five females were judiciously selected from their rearing environment. These insects, aged up to 24 hours postseed emergence, were deposited into each plate, and the plates were promptly resealed postintroduction. This carefully orchestrated experimental design was systematically replicated three times for each concentration, adhering to stringent research practices [18].

Over a span of four consecutive days, at 24-hour intervals, a meticulous count of deceased insects was systematically recorded to discern and evaluate the mortality rate within the respective concentration levels. Additionally, a thorough examination of eggs laid on the seeds and plate walls was conducted, employing a binocular magnifying glass for enhanced precision. Furthermore, a consistent count of insects emerged was conducted commencing from the 28th day following their initial confinement. These detailed observations and counts were systematically juxtaposed with those from the control groups, forming the basis for determining reduction rates in both eggs laid and emergence [18].

The mortality rate, corrected by Abbott's formula, was computed through the expression:

(2) $$\begin{array}{c} \text{mortality }\left(\%\right)=100\times \frac{Po-Pc}{100-Pc}, \end{array}$$

where *Po* and *Pc* represent the observed mortality in the test and control, respectively.

The egg-laying reduction rate was calculated using the following formula:

(3) $$\begin{array}{c} \text{reduction of egg laying }\left(\%\right)=100\times \frac{Nc-Nt}{Nc}, \end{array}$$

where *Nt* and *Nc* represent the number of eggs in the test and control jars, respectively.

The reduction rate of emerged insects was determined using the formula as follows:

(4) $$\begin{array}{c} \text{reduction of emergence }\left(\%\right)=100\times \frac{Nc-Nt}{Nc}, \end{array}$$

where *Nc* and *Nt* signify the number of insects hatched in the control and test jars, respectively. This meticulous experimental framework, coupled with a rigorous analytical approach, aims to contribute valuable insights into the field of insecticidal research, specifically in understanding the impact of *Rosmarinus officinalis* EO on *Callosobruchus maculatus*.

*(2) Toxicity of the Essential Oil by Inhalation*. In summary, the experimental setup involved suspending miniature cotton loops within glass jars, to which varying concentrations of *Rosmarinus officinalis* essential oil (1 *μ*L, 5 *μ*L, 10 *μ*L, and 20 *μ*L) were meticulously applied using a micropipette. Subsequently, a total of 10 *Callosobruchus maculatus* bruchids (comprising both males and females) aged no more than 48 hours were introduced into each jar. The jars were then hermetically sealed to create a controlled environment. Three replicates were meticulously conducted for each experimental condition. To provide a baseline for comparison, a control group was established, consisting of cotton loops without the essential oil solution. The subsequent computation of the mortality rate was performed using previously described and validated methodologies. This experimental design aimed to systematically evaluate the impact of varying concentrations of *Rosmarinus officinalis* EO on the mortality of *Callosobruchus maculatus*, contributing to a nuanced understanding of its insecticidal efficacy [18].

#### 2.5.3. Essential Oil's Repellent Activity against *C. maculatus*

In this investigative study, the evaluation of the repellent properties demonstrated by the essential oil derived from *R. officinalis* against *C*. *maculatus* insects was conducted utilizing the preferential area method, as elucidated in previous scholarly research [8]. The experimental protocol involved the strategic placement of filter paper disks within Petri plates. Each disk (9 cm of diameter) was meticulously divided into equal halves, resulting in sections measuring 31.80 cm^2^ each. The halves were subjected to different treatments: one received a uniform application of 0.5 mL of varying EO concentrations (5, 10, and 20 *μ*L/mL) prepared in acetone, corresponding to doses of 0.079, 0.157, and 0.315 *μ*L/cm^2^ per disk, respectively, while the other half was exclusively impregnated with 0.5 mL of acetone. Thereafter, 10 pairs of *C*. *maculatus* adult bruchids, each less than a day old, were carefully positioned in the central region of each Petri plate. To maintain a controlled environment, the plates were securely sealed using parafilm. To ensure the robustness and reliability of the results, each experimental run was meticulously repeated three times, ensuring consistency with the conditions prevailing in the insect-rearing environment.

Following a precisely controlled 30-minute exposure period, the enumeration of bruchids present on the EO-treated section of the disk was conducted and meticulously compared with the control treated solely with acetone. The repulsion percentage, a key metric in assessing the efficacy of the EO as a repellent, was calculated using the formula [15]:

(5) $$\begin{array}{c} \text{repulsion }\left(\%\right)=\frac{\text{NC}-\text{NT}}{\text{NC}+\text{NT}}\times 100, \end{array}$$

where NC and NT represent the numbers of *C*. *maculatus* insects in the control zone and treated zone, respectively. This quantitative approach facilitated a comprehensive evaluation of the repellent effectiveness of *R. officinalis* EO against *Callosobruchus maculatus.*

Furthermore, the obtained average repellency percentage was subjected to categorization into specific repellency classes ranging from 0 to 100% [19]. This classification provided a nuanced understanding of the repellent potential of *R. officinalis* EO, contributing to the broader knowledge base concerning its applications in insect management. The systematic and thorough nature of this experimental design enhances the reliability and applicability of the findings, thereby advancing our comprehension of the insect-repelling attributes of *R*. *officinalis* EO.

### 2.6. *In Silico* Molecular Docking of Essential Oil's Antifungal and Insecticidal Activities

In this research, the utilization of computational methods aimed to evaluate the biological activities of *R. officinalis* EO involved a comprehensive investigation into the inhibition of acetylcholinesterase for assessing insecticidal potential and the inhibition of *Botrytis cinerea β*-tubulin to evaluate antifungal activity. For the preparation of ligands, we meticulously compiled all the compounds identified in *R. officinalis* EO through GC/MS from PUBCHEM in Structure Data File (SDF) format. Subsequently, these diverse ligands were subjected to a complete pretreatment phase for the docking calculations using the LigPrep tool within the Schrödinger Software program (v. 11.5). This process involved applying the OPLS3 force field, the generation of up to 32 stereoisomers for each ligand, and specification of ionization states at pH 7.0 ± 2.0 [9].

The preparation of proteins involved obtaining three-dimensional crystal structures of acetylcholinesterase (PDB: 6ARY) [20] and *Botrytis cinerea β*-tubulin (PDB: 3N2G) [21] from the Protein Data Bank (PDB) in PDB format. These structures underwent meticulous construction and refinement by the use of the Protein Preparation Wizard in Schrödinger-Maestro (v. 11.5). The process involved introducing hydrogens (H) to heavy atoms, converting selenomethionines to methionines, and eliminating all water molecules. Following these steps, the proteins underwent minimization utilizing the OPLS3 force field, with the maximum. The creation of the receptor grid commenced with the initiation of the creation module, wherein a ligand atom was chosen, resulting to the establishment of a default grid box. Subsequently, the ligand was connected to the grid box generated from the protein by the use of the Standard Precision.

Executing the Standard Precision, flexible ligand docking procedure was conducted through the Glide module within Schrödinger-Maestro (v. 11.5). This involved incorporating penalties for non-*cis/trans* amide bonds. Specific parameters for ligand atoms, such as the Van der Waals scaling factor and the partial charge cutoff, were carefully programmed to 0.80 and 0.15, respectively. The final score, determined based on energy-minimized poses, was presented as a Glide score. The optimal docked pose for each ligand was identified as the one with the lowest Glide score value [22]. This rigorous computational approach aimed to unveil the potential molecular interactions of *Rosmarinus officinalis* EO compounds with acetylcholinesterase and *Botrytis cinerea β*-tubulin, providing valuable insights into their insecticidal and antifungal mechanisms of action.

### 2.7. Statistical Analysis

The computation of mean values and standard deviations (SD) was carried out using GraphPad Prism 8, a software developed by Microsoft. The antifungal and insecticidal test results were statistically analyzed using the same software platform. A one-way ANOVA and a Tukey test were then performed. A statistically significant difference between groups was shown by a result below the predetermined significance level of *p* < 0.05. Moreover, lethal concentrations (LC_50_ and LC_95_), along with their corresponding confidence intervals, were calculated through the application of the probit method [23], after fitting a probit model to the data obtained. Quantile functions corresponding to probabilities of 0.50 and 0.95, respectively, were used to calculate LC_50_ and LC_95_ doses, in order to assess the degree of efficacy of the rosemary EO studied. These statistical analyses were instrumental in providing a robust and comprehensive evaluation of the experimental data, facilitating meaningful comparisons and inferences drawn from the study.

## 3. Results and Discussion

### 3.1. Essential Oil Yield

The leaves of *R. officinalis* collected from Boulemane (Morocco), produced a clear yellow EO possessing a distinctive aroma, and rendered a yield of 2.44% (*v/w*). It is widely recognized that the yield of EO is influenced by several factors, including the plant species, the geographical distribution, and the organ used. The collection period and the extraction method were also involved [24]. For instance, the EOs extracted from *R. officinalis* leaves in studies conducted by El-Demerdash et al. Al Zuhairi et al. and Rezouki et al. yielded 3.2%, 0.98%, and 1.25%, respectively [25–27]. This discrepancy in yield is often due to the geographic origin of the plant collected [9].

### 3.2. Essential Oil's Phytochemical Composition

The EO extracted from *R*. *officinalis* underwent thorough examination and identification of its chemical composition through the utilization of GC-MS technique. The identified constituents are comprehensively detailed in Table 1. According to the data obtained, a total of sixteen compounds were discerned in the *Rosmarinus officinalis* EO, collectively representing 98.82% of the total essence. Predominant among these compounds were 1,8-cineole (40.80%), *α*-pinene (26.18%), and camphor (19.53%), all belonging to the class of monoterpenes. Notably, these monoterpenes constituted the major components of the investigated EO. Other compounds were present in yields of less than 3%. Comparative studies on EO extracted from Spanish *R*. *officinalis* leaves revealed a high content of 1,8-cineole (26.12%) and camphor (15.81%) [28]. Similarly, in an investigation conducted by El-Demerdash et al. in 2021, *α*-pinene (13.64%) along with 1,8-cineole (41.75%) and camphor (17.66%) was identified as major components in *R. officinalis* EO [27]. Furthermore, 1,8-cineole (17.16%) emerged as a major component in rosemary EO collected from Iran [24]. Caputo et al. (2018) discovered that the primary compounds of the Italian *R. officinalis* EO consisted of *α*-pinene, verbenol, verbenone, 1,8-cineol, and isoborneol [29]. Likewise, Jangwan and collaborators, in 2016, pinpointed germacrene-D, isoeugenol, heneicosene, 9-nonadecene, geraniol, *α*-pinene, tricosane, *β*-caryophyllene, and heptacosane as the predominant compounds [30]. Kaab et al. documented 1,8-cineole, camphor, and *α*-pinene as the principal constituents, while Ngan et al. detected *α*-pinene, eucalyptol, camphene, bicyclo[3.1.1]hept-3-en-2-one, caryophyllene, endo-borneol, and bornyl acetate as the primary components of rosemary EO [31, 32]. These variations in compound composition are likely attributed to seasonal fluctuations, environmental factors, and circadian rhythms [24]. Importantly, several compounds identified in these EOs, including 1,8-cineole, linalool, camphor, and borneol, are recognized for their potential bioactivity and diverse pharmacological properties [33]. Moreover, constituents such as linalool, borneol, 1,8-cineole, *α*-pinene, and camphor identified in the examined essential oil of *R. officinalis* have been noted to demonstrate antifungal properties against a wide range of fungi [12, 34, 35]. Otherwise, compounds such as camphor, 1,8-cineole, and R-(+)-limonene, which were identified by EO, have also demonstrated efficacy against agricultural pests [36–39]. The multifaceted chemical composition of *R*. *officinalis* EO underscores its potential as a source of bioactive compounds, further substantiating its significance in various applications within the realms of pharmacology and natural products research.

### 3.3. Essential Oil's Antifungal Activity

**Figure 1:**
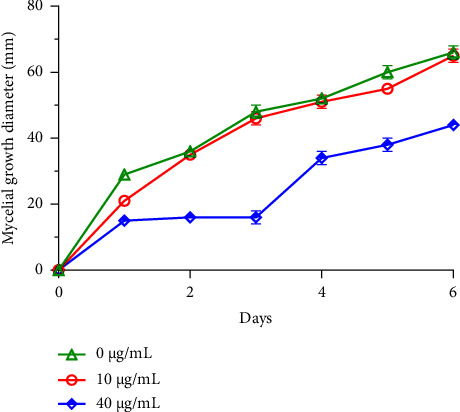
Mycelial growth of *A. alternata* with time, following treatment with two concentrations of *R. officinalis* EO.

**Figure 2:**
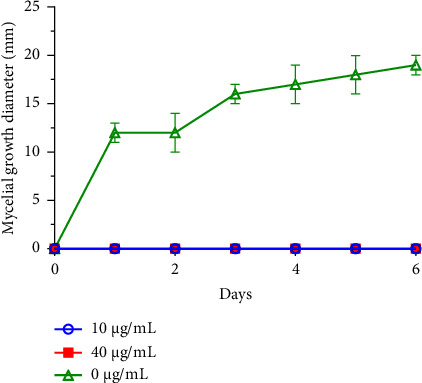
Mycelial growth of *B. cinerea* with time, following treatment with two concentrations of *R. officinalis* EO.

**Figure 3:**
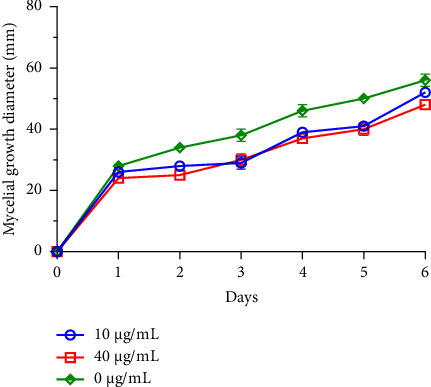
Mycelial growth of *F. oxysporum* with time, following treatment with two concentrations of *R. officinalis* EO.

Filamentous fungi, in particular species of the genera *Fusarium*, *Alternaria*, and *Botrytis*, are commonly associated with the contamination of legume and fruit crops during harvesting and storage, thus constituting a significant threat [40]. These molds, known for their capacity to produce mycotoxins, are dangerous and pathogenic for humans [5]. In the context of this investigation, two distinct concentrations of *R*. *officinalis* EO underwent testing to assess their, *in vitro*, antifungal effects against *B. cinerea*, *F. oxysporum*, and *A. alternata* growths (Figures 1, 2, and 3).

The results revealed pronounced antifungal effects of rosemary EO against the investigated fungi, displaying a dose-dependent inhibition of their mycelial growth. Throughout the entire incubation period, *R. officinalis* oil completely inhibited *B. cinerea* mycelial growth across all tested concentrations. Furthermore, the concentration of 10 *μ*g/mL of oil was ineffective against *A. alternata* mycelial growth, while the 40 *μ*g/mL concentration demonstrated partial antifungal activity. In the case of *F. oxysporum*, both concentrations, with particular emphasis on the 40 *μ*g/mL concentration, demonstrated inhibitory effects compared to the control. These effects were noted in terms of delayed kinetics of fungal mycelial growth, which commenced on the first day of observation.

These findings underscore the potential of *R*. *officinalis* EO as an effective bio-antifungal agent against significant plant-pathogenic fungi, suggesting its utility in mitigating the risks associated with fungal contamination in crops, particularly those caused by *B. cinerea, F. oxysporum,* and *A. alternata.*

**Figure 4:**
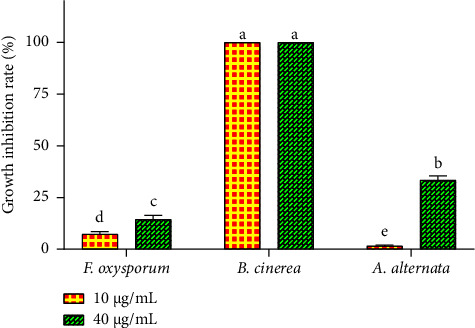
Reduction in the growth rate (%) of *A. alternata, B. cinerea*, and *F. oxysporum* after the sixth day of treatment with two concentrations of *R. officinalis* EO. Value bars with different letters are significantly different (*p* < 0.05).

The growth inhibition rate (%) of the three fungal strains tested was evaluated during the 6^th^ day of incubation for both concentrations of *R. officinalis* EO. The outcomes depicted in Figure 4 indicate that *B. cinerea* displayed the highest sensitivity to rosemary EO, with its growth completely inhibited (100%) by both concentrations of the oil. In contrast, *F. oxysporum* and *A. alternata* exhibited lower sensitivity to the essential oil. The 40 *μ*g/mL concentration of the oil resulted in a partial inhibition of *A. alternata* growth, reaching 33.43 ± 2.01% after 6 days of incubation. Similarly, the same oil concentration led to a 14.38 ± 1.98% inhibition of *F. oxysporum* mycelial growth over the same incubation period.

Our results align with a recent investigation, which demonstrated that *R. officinalis* EO, containing primarily 1,8-cineole (52.20%), camphor (15.20%), and *α*-pinene (12.40%), displayed a reduction of 15.3% in *Aspergillus flavus* mycelial growth when used at 250 *μ*g/mL. The study also revealed minimum inhibitory and fungicidal concentrations of 500 *μ*g/mL for the same fungal strain. Additionally, the *R. officinalis* EO exhibited an anti-aflatoxigenic effect by inhibiting the synthesis of aflatoxins B_1_ and B_2_ after treatment with 250 *μ*g/mL concentration [41]. Additionally, the study reported alterations in the morphology of *A. flavus*, such as reduced conidiophore size and hyphal thickness when treated with *R. officinalis* EO (250 *μ*g/mL) as observed through scanning electron microscopy [41]. Moreover, our findings surpassed those reported by Šernaitė et al. where application of *R. officinalis* EO at a concentration of 2000 *μ*l/mL reduced *B. cinerea* mycelial growth by 31.91% [42]. Other study reported that the minimum inhibitory concentration (MIC) observed when rosemary EO was applied against *Fusarium oxysporum* f.sp. *Albedinis* was 0.2 g/L [43].

The antifungal efficacy of EOs is often linked to their chemical composition, potentially due to the individual or synergistic effects of major and minor components. Compounds like linalool, borneol, 1,8-cineole, and camphor detected in the studied *R. officinalis* EO could contribute to its observed fungicidal activity. Studies have highlighted the potency of linalool, followed by 1,8-cineole, against *A. pullulans*, *D. hansenii*, and the genus *Penicillium*, exhibiting strong inhibitory effects on their mycelial growth [34]. Moreover, *in vitro* assessments have indicated a significant reduction in *A. alternata* mycelial growth upon exposure to chemical compounds found in rosemary oil, such as camphor, *α*-pinene, and borneol [35]. Additionally, various monoterpenes present in rosemary EO, including limonene and 1,8-cineole, have demonstrated antifungal properties [12]. Therefore, the wide range of compounds present in the investigated EO, coupled with their diverse antifungal capacities, poses challenges in identifying the specific active components responsible for the antifungal action.

Various mechanisms have been proposed to elucidate the toxic effect of EOs against fungi. For instance, the antifungal effects of rosemary may be associated with the terpenes and phenolic compounds in the EO. These components are recognized for their ability to disrupt cell membranes, leading to the leakage of cellular contents, hindering electron transport, ATPase activity within mitochondria, and ultimately resulting in microbial death [44]. In related studies, certain EOs were noted to significantly diminish the synthesis of phospholipase enzymes in *C. albicans* strains, thereby substantially reducing their virulence [45].

### 3.4. Essential Oil's Insecticidal Activity

#### 3.4.1. Essential Oil's Toxicity against *C. maculatus*

**Figure 5:**
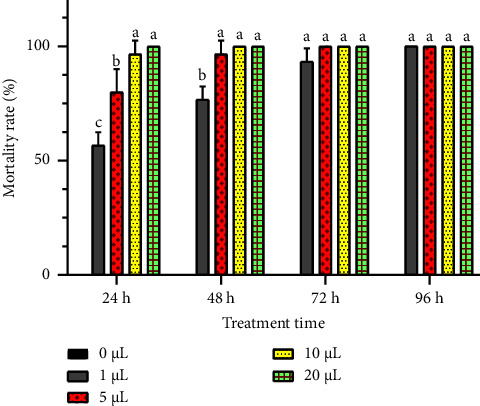
Mortality rates (means ± SD) of *C. maculatus* adults when exposed to different concentrations of *R. officinalis* EO in a contact toxicity test. Value bars with different letters are significantly different (*p* < 0.05).

**Figure 6:**
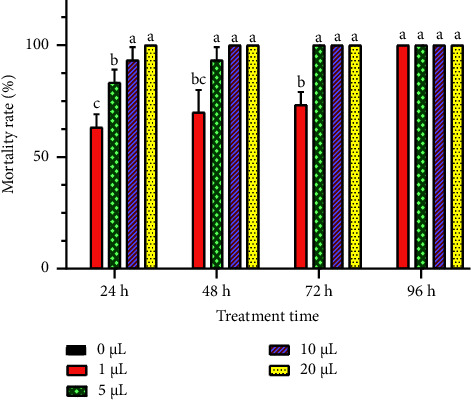
Mortality rates (means ± SD) of *C. maculatus* adults when exposed to different concentrations of *R. officinalis* EO in an inhalation toxicity test. Value bars with different letters are significantly different (*p* < 0.05).

The toxicity of *R. officinalis* EO against *C. maculatus* insect was assessed via two various tests, focusing on inhalation and contact, to evaluate its insecticidal capacity against this famous chickpea pest. Results, detailed in Figures 5 and 6, revealed a significant insecticidal effect of the studied rosemary EO in both contact and inhalation tests. The mortality of *C. maculatus* adults increased with higher EO doses and prolonged exposure durations. At the lowest concentration (1 *μ*L/L), *R. officinalis* EO induced mortality rates of 56.66 ± 5.77% and 63.33 ± 5.77% in *C. maculatus* adults after 24 hours in the contact and inhalation tests, respectively. Over time, these rates significantly escalated (*p* < 0.05) with exposure duration, resulting in total insect mortality after 96 hours. Notably, at higher concentrations (10 *μ*L/L and 20 *μ*L/L), complete mortality (100%) was noted within the initial 24 hours in chickpea bruchid adults exposed to the EO in both tests. The control jar showed a mortality rate of 0%.

The lethal concentrations (LC_50_ and LC_95_) of rosemary EO causing 50% and 95% mortality, respectively, in *C. maculatus* adults within 24 hours were determined and are listed in Table 2. The findings indicate a more pronounced biocidal effect in the inhalation test, evidenced by a lower LC_50_ value (0.62 *μ*L/L air) compared to the contact test (LC_50_ = 1.15 *μ*L/L air). This observation was also confirmed by LC_95_ data (Table 2).

#### 3.4.2. Essential Oil's Effect on Fecundity and Emergence of *C. maculatus* Individuals

**Figure 7:**
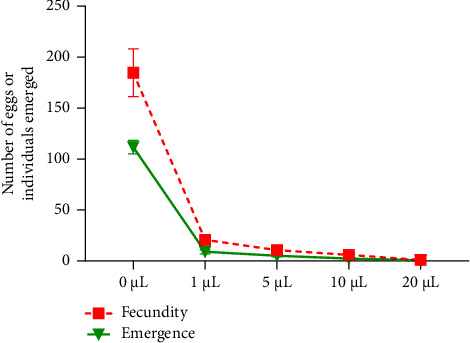
Female fecundity (mean number of eggs laid ± SD) and adult emergence (mean ± SD) of *C. maculatus* following a direct contact toxicity test with different concentrations of *R. officinalis* EO.

**Figure 8:**
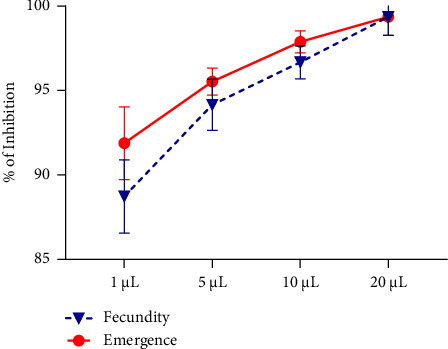
Fecundity and emergence inhibition (%) of *C. maculatus* adults after a toxicity test by direct contact with different concentrations of *R. officinalis* EO.

The influence of *R*. *officinalis* EO on the oviposition behavior and subsequent emergence of new *C*. *maculatus* individuals were systematically investigated, and the findings are presented in Figures 7 and 8. The results reveal a conspicuous inverse correlation between EO concentration and the number of eggs laid. At the lowest concentration (1 *μ*L/L), the average oviposition decreased to 20.66 ± 4.04 eggs per female, representing a remarkable fecundity reduction rate of 91.88% relative to the control. At the highest concentration (20 *μ*L/L), the average number of eggs laid/female significantly decreased to 1.00 ± 1.73 eggs, indicating a profound 99.36% reduction in oviposition. In the control jar, *C. maculatus* females laid an average of 184.66 ± 23.43 eggs per female.

Conversely, the number of emergences displayed a noteworthy decline with increasing EO concentrations, as depicted in Figures 7 and 8. At the lowest EO concentration (1 *μ*L/L), the emergent *C. maculatus* larvae, after embryonic development in chickpea seeds, numbered 9.00 ± 2.00 individuals, compared to 111.66 ± 6.50 individuals in the control. This reflected a substantial emergence inhibition rate of 91.88%. Meanwhile, at the highest concentration (20 *μ*L/L), the studied EO exerted a profound inhibitory effect on the emergence of new individuals, resulting in a remarkable reduction of 99.36%.

These findings underscore the potent impact of *R*. *officinalis* EO on the reproductive behavior and subsequent emergence of *C*. *maculatus*, suggesting its potential application as an effective biopesticide for controlling insect infestation in stored chickpea seeds.

#### 3.4.3. Repellent Activity of Essential Oil against *C. maculatus*

In addition to its biocidal efficacy, the repellent potential of *R*. *officinalis* EO against *C. maculatus* insects was systematically evaluated, and the results are listed in Table 3. Overall, the various concentrations of rosemary EO tested exhibited a moderate repellent effect, as per McDonald's (1970) classification [46]. The observed rate of repellence displayed a dose-dependent relationship, reaching a maximum of 80.00% after 30 minutes of exposure to the highest concentration of 0.315 *μ*L/cm^2^ EO. The average percentage of repellence for the same duration of treatment was determined to be 50.83%.

These findings highlight the noteworthy repellent properties of *R*. *officinalis* essential oil, signifying its potential utility in deterring and mitigating *C*. *maculatus* infestation. The dose-dependent nature of the repellent effect further suggests the feasibility of utilizing varying concentrations of rosemary EO to optimize its repellent efficacy for effective insect management.

The insecticidal activity of *R. officinalis* EO on *Callosobruchus maculatus* was studied in this work, revealing significant effects on this chickpea pest. The results of these studies show a remarkable ability of rosemary essential oil to induce mortality in *C. maculatus* adults, while also affecting their fecundity and emergence. Inhalation and contact tests demonstrated a progressive increase in mortality with higher doses of EO and prolonged exposure times. For example, at lower concentrations, partial mortality was observed after 24 hours, while prolonged exposure resulted in total insect mortality. In addition, this study revealed that rosemary EO also had a significant impact on insect fecundity, reducing their ability to reproduce effectively. The same EO also had a significant impact on insect fecundity, reducing their ability to reproduce effectively. Moreover, higher concentrations of the studied *R. officinalis* EO led to more rapid mortality, with complete elimination of insects within the first few hours of exposure.

Our findings align with Ainane et al. [47] research, which demonstrated a strong toxic effect of *R. officinalis* EO against *T. confusum* resulting in total insect mortality at a dose of 12 × 10^−2 ^*μ*l/cm^3^ after one day of exposure to the treatment. Similarly, recent studies reported the insecticide activity of the rosemary EO against many stored product pests, including *C*. *maculatus*, *T*. *castaneum*, *S*. *granaries*, *S*. *oryzae*, and *T*. *confusum*. In the same approach, investigations into *L. dentata* and *O. compactum* EOs belonging to the same family of *R. officinalis* (Lamiaceae) revealed significant toxic action against the studied *C. maculatus* insect marking notable LC_50_ value of 4.01 *μ*L/L air and 5.3 *μ*L/L air, respectively [8, 15]. These same EOs exhibited complete inhibition (100%) of oviposition and emergence of the insect at higher concentrations (20 *μ*L/L air). Generally, the insecticidal effect of *R. officinalis* EO against the tested pest can be attributed to its main components, in particular, camphor and eucalyptol (1,8-cineole), which showed strong insecticidal activity against *S. zeamais* and *T. castaneum* insects of stored legume and cereal seeds [36, 37]. In addition, the 1,8-cineole and R-(+)-limonene are insecticidal, mainly in the ingestion and/or contact test, against two insects (*R. dominica* and *T. castaneum*), which lead to significant economic losses of stored wheat grain [11]. In the same sense, other reports have noted that EOs rich in eucalyptol or camphor are highly toxic for phytophagous insects in general [38, 39].

Although this article did not investigate the precise mechanism of action of rosemary EO toxicity on insects, previous studies have looked at the effects of terpenoids, such as 1,8-cineole and camphor. These compounds were found to have an impact on the nervous system by inhibiting the activity of the enzyme acetylcholinesterase (AChE) in insects [48–51]. Moreover, a study evaluating the effect of *M. arvensis* EO on *S. granarius* adults after contact reported rapid paralysis in addition to an altered walking behavior in the pests after treatment [19, 36]. This study also noted significant physiological changes caused by the essences of *M. arvensis* in the treated insects, resulting in an upregulation of various differentially expressed proteins (DEPs). These proteins play a role in the development and functioning of the nervous and muscular systems, cellular respiration, protein synthesis, and detoxification [52]. These results underline the considerable influence of EO on various biological processes and elucidate the mechanisms utilized by surviving insects to recover from damage.

A remarkable decrease in both fecundity and emergence rates was evident in the *C. maculatus* insect, indicating the potent ovicidal and larvicidal effects of *R. officinalis* EO studied. This observed ovicidal effect of the EO tested could be attributed to the hindering of embryonic development following penetration of oil vapors into the eggs via the respiratory tract of the *C. maculatus* insect, as suggested by several researchers [53, 54]. This phenomenon is thought to be linked to the direct toxicity of EO components, which inhibit or limit metabolic activity inside the eggs. This effect was observed with compounds such as piperitone, isolated from *C. schoenanthus* EO tested on *C. maculatus* eggs [55], and ß-asarone, identified in *A. calamus* EO applicated on *C. chinensis, S. oryzae,* and *S. granarius* eggs [56]. Furthermore, research conducted on another bruchid species revealed variations in the sensitivity and vulnerability of eggs to the vapors of three EOs, including *R. officinalis*, depending on egg age and stage of embryonic development [57]. Moreover, some investigations have highlighted the sterilizing effect of EOs on eggs [56].

Our investigation revealed a substantial decrease in emergence rates, evident even at the 1 *μ*L/L dose of rosemary EO. This reduction might be attributable to the larvicidal effects of EO's monoterpenic compounds. A similar finding was supported by the work of Kheloul and colleagues, who noted that young larvae (L_1_) of *T. confusum*, a cereal seed pest, exhibited heightened sensitivity to the toxic impact of both *L. spica* EO and linalool (compound of the studied EO). They reported LC_50_ values of 19.535 *μ*L/L air and 14.198 *μ*L/L air, respectively, after 24 hours of exposure time [58]. Additionally, their study revealed that linalool induced greater egg mortality compared to *L. spica* EO at the same concentrations, consequently reducing the emergence of surviving adults, larvae, and pupae [58].

The repellent effect observed with *R. officinalis* EO appeared moderate across all tested concentrations. Typically, the effectiveness of repellents derived from EOs is transient, largely owing to their volatility. In comparison, synthetic repellents often demonstrate greater efficacy and persistence than natural counterparts [59]. The duration of repellence exhibited by EOs is primarily influenced by their chemical composition. Many monoterpenes like camphor, *α*-pinene, and cineole, commonly found in various EOs, have been highlighted in the literature for their significant mosquito-repellent properties, as reported by some researchers [60, 61].

Overall, the findings of the current study underline the strong insecticidal potential of the examined EO in the control of *C. maculatus* insect and suggest that it could be used as an effective alternative to synthetic chemicals in the protection of chickpea crops. However, further studies are needed to deepen our understanding of the mechanisms of action involved, including their impact on insect fecundity and emergence, as well as to assess the impact of these essential oils on other nontarget organisms and on the environment as a whole.

Given the known functions of EOs in suppressing fungi, bacteria, viruses, pests, and weeds, several studies have reported their potential applications in agriculture at low doses or in combination with conventional insecticides and fungicides [62]. Recently, there have been promising results using nanoformulations or bioinformatics tools [63–65]. However, their high volatility, low stability, poor water solubility, significant influence on organoleptic properties, and phytotoxic effects remain obstacles to overcome in the hope of developing biofungicides and bioinsecticides derived from EOs [62].

### 3.5. *In Silico* Molecular Docking of Essential Oil's Antifungal and Insecticidal Activities

In contemporary research endeavors, molecular modeling has become an essential tool, using computer-aided drug design (CADD) technologies to study and predict the interactions of various compounds, whether stable or volatile, such as essential oils (HE), with molecular targets linked to various biological activities [66]. In the current research, molecular docking was employed to assess the inhibitory action of compounds present in *R*. *officinalis* EO against *β*-tubulin, a key component in the vitality of *Botrytis cinerea*. *β*-tubulin, in association with *α*-tubulin, forms microtubules, crucial components of the cytoskeleton. Microtubules play pivotal roles in cellular processes, including maintaining cell structure, facilitating cell division, and influencing fungal virulence [21]. *In silico* evaluation of the antifungal activity revealed that *α*-caryophyllene, *β*-caryophyllene, and verbenone, constituents of *R. officinalis* EO, exhibited significant antifungal activity against *Botrytis cinerea β*-tubulin (PDB: 3N2G), with scores of −7.025, −6.772, and −6.213 kcal/mol, respectively (Table 4). These *in silico* data confirm the antifungal properties of caryophyllene, as reported in previous studies [67].

**Figure 9:**
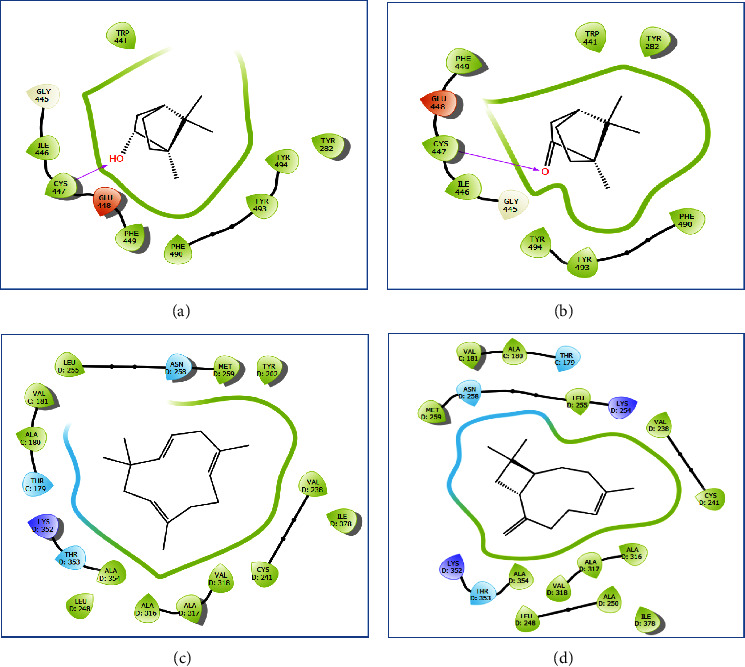
The 2D viewer of ligand interactions with the active site. (a, b) Borneol and camphor interactions with the active site of acetylcholinesterase; (c, d) *α*-caryophyllene and *β*-caryophyllene interactions with active site of sterol *Botrytis cinerea β*-tubulin.

**Figure 10:**
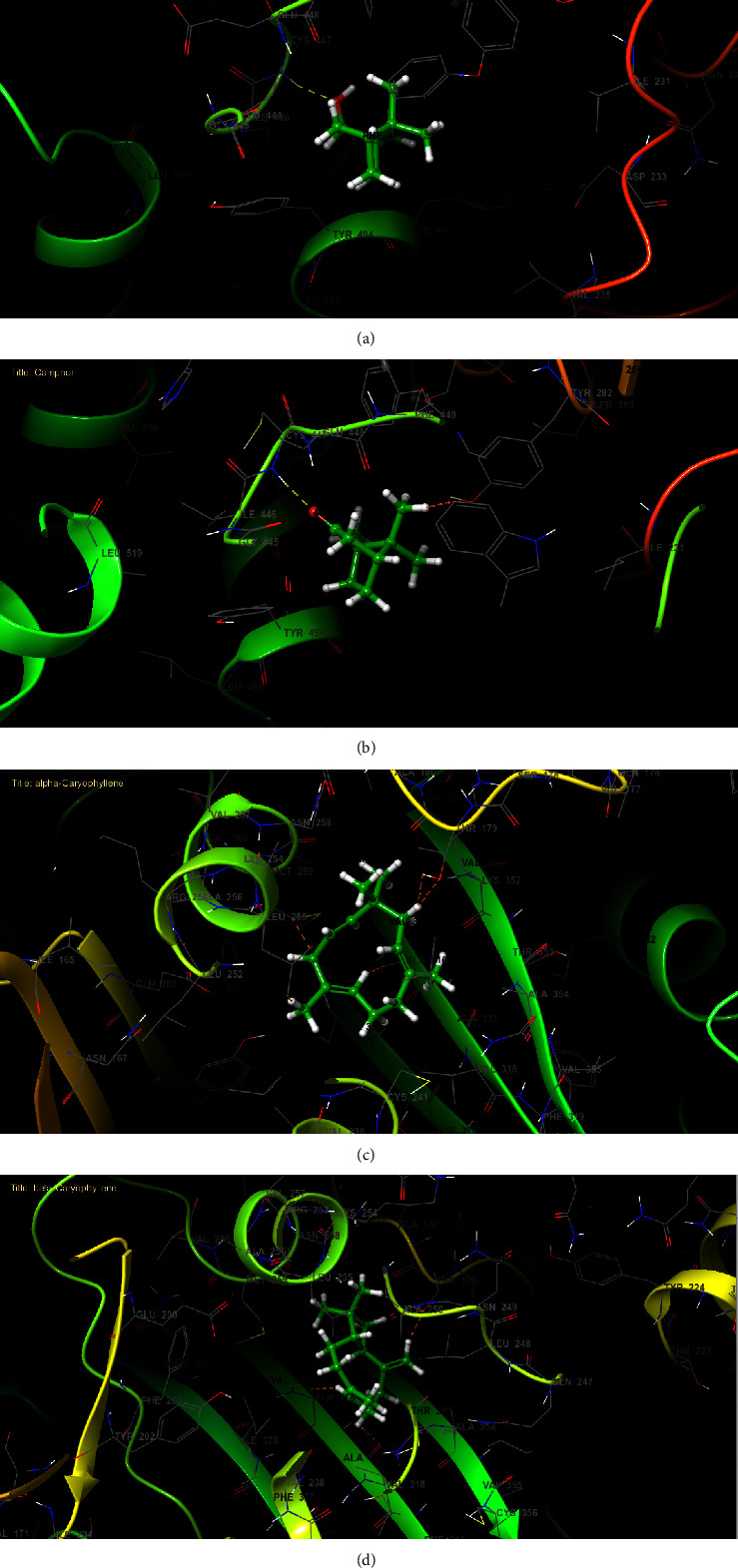
The 3D viewer of ligand interactions with the active site. (a, b) Borneol and camphor interactions with the active site of acetylcholinesterase; (c, d) *α*-caryophyllene and *β*-caryophyllene interactions with the active site of *Botrytis cinerea β*-tubulin.

On another front, acetylcholinesterase is a pivotal enzyme for the proper functioning of the insect nervous system. Consequently, its inhibition is a critical factor in achieving insecticidal effects. *In silico* data pertaining to *R. officinalis* EO indicated that borneol, camphor, and *α*-caryophyllene were the molecules displaying the highest effectiveness against acetylcholinesterase (PDB: 6ARY), with Glide scores of −7.254, −7.023, and −6.276 kcal/mol, respectively. These findings affirm the insecticidal impact of several monoterpenes identified in the studied rosemary EO and their influence on the nervous system by inhibiting the activity of the acetylcholinesterase enzyme in various insects [49–51]. Moreover, 2D and 3D viewers of *R. officinalis* EO docked in the active site of acetylcholinesterase (PDB: 6ARY) revealed that borneol established a single hydrogen bond with residue CYS 447 (Figures 9(a) and 10(a)). Similarly, camphor also formed a hydrogen bond with the same residue in the same active site (Figures 9(b) and 10(b)).

Previous studies have examined the way by which EOs act on insects, revealing in particular their impact on the enzyme acetylcholinesterase. This enzyme plays a key role in the insect nervous system, being responsible for the degradation of acetylcholine, a neurotransmitter crucial to the transmission of nerve impulses. The action of EOs on acetylcholinesterase can disrupt this process, leading to neurological dysfunction in insects. This disruption can result in paralysis or rapid death of the insects, making it an important mechanism in the insecticidal activity of essential oils. However, it should be noted that EOs can also act on other biological targets, such as ion channels or respiratory systems [68–70]. Our results indicate that the molecule with the highest affinity for acetylcholinesterase is *α*-caryophyllene. Other experimental and theoretical works on other insects have also confirmed the anti-acetylcholinesterase effect of *α*- and *β*-caryophyllene [71, 72]. Other compounds, such as 1,8-cineole [73–75], *α*-pinene [76], and *δ*- and *γ*-cadinene [77], present in EOs, have also demonstrated acetylcholinesterase-inhibiting activity. EOs composed of a mixture of bioactive molecules, mainly sesquiterpenes and monoterpenes, appear to have a more pronounced acetylcholinesterase inhibitory effect. It is possible that the synergistic combination of these molecules is responsible for their inhibitory action.

These molecular docking results provide valuable insights into the potential mechanisms underlying the antifungal and insecticidal activities of specific compounds in *Rosmarinus officinalis* EO, contributing to a comprehensive understanding of their bioactive properties at the molecular level.

## 4. Conclusion

In summary, the results unequivocally demonstrate that the EO derived from Moroccan *Rosmarinus officinalis* exhibits robust antifungal and insecticidal properties. These experimental findings are corroborated by *in silico* analyses, which confirm the inhibitory effect of rosemary EO on insect acetylcholinesterase and fungal *β*-tubulin. Accordingly, these conclusions underscore its potential utility as an efficacious antifungal and bioinsecticidal agent for agricultural crops and the storage of leguminous products. The observed biological activities can be attributed, at least in part, to the presence of monoterpene compounds within the EO. The multifaceted benefits of *Rosmarinus officinalis* EO, as elucidated through this study, provide a solid foundation for further exploration and development of its practical applications in crop protection and storage management.

## Figures and Tables

**Table 1:** Phytochemical compounds of the EO extracted from *R. officinalis*.

Compounds	RI	MW	Compounds	Molecular formula	Content (%)
1	939	136	*α*-Pinene	C_10_H_16_	26.18
2	954	136	Camphene	C_10_H_16_	2.23
3	979	136	*β*-Pinene	C_10_H_16_	0.39
4	1017	136	*α*-Terpinene	C_10_H_16_	0.53
5	1025	134	*p*-Cymene	C_10_H_14_	1.30
6	1028	136	Limonene	C_10_H_16_	2.71
7	1030	154	1,8-Cineole	C_10_H_18_O	40.80
8	1048	154	*β*-Myrcene	C_10_H_16_	2.11
9	1097	154	Linalool	C_10_H_18_O	0.18
10	1146	152	Camphor	C_10_H_16_O	19.53
11	1169	154	Borneol	C_10_H_18_O	0.52
12	1199	154	*α*-Terpineol	C_10_H_18_O	1.26
13	1205	150	Verbenone	C_10_H_14_O	0.75
14	1289	196	Bornyl acetate	C_12_H_20_O_2_	0.04
15	1419	204	*β*-Caryophyllene	C_15_H_24_	0.11
16	1423	204	*α*-Caryophyllene	C_15_H_24_	0.18
	Total (%)				98.82

RI: retention index; MW: molecular weight.

**Table 2:** Lethal concentrations of LC_50_ and LC_95_ (*μ*L/L of air) induced mortality in *C. maculatus* adults in contact and inhalation toxicity tests after 24 h of treatment with *R. officinalis* EO.

Bioassays	df	Slope ± SD	LC_50_ (CI 95%)	LC_95_ (CI 95%)	Intercept ± SD	*p* Value	*χ* ^2^
Inhalation	2	1.3 ± 0.18	0.62 (0.0; 1.93)	11.51 (4.00; 1.98 *∗* 10^11^)	0.27 ± 0.12	0.00	5.084
Contact	2	1.56 ± 0.18	1.15 (0.0; 3.10)	13.08 (4.61; 5.37 *∗* 10^8^)	−0.09 ± 0.1	0.00	7.34

The chi-square test (*χ*^2^) was used to determine the slope of a line. Slope is determined by Probit (*p*) = constant + Bx (covariates × transformed using log base 10); df: degrees of freedom; SD: standard deviation; LC50 and LC_95_ lethal concentrations (causing 50% and 95% mortality of C. *maculatus* adults); CI: 95% confidence interval.

**Table 3:** Results of the repellent activity of R. officinalis EO against C. maculatus after 30 min of treatment.

Repellent activity (%) at different doses of EO				PR average (%)	Class^*∗*^
0.039 (*μ*L/cm^2^)	0.079 (*μ*L/cm^2^)	0.157 (*μ*L/cm^2^)	0.315 (*μ*L/cm^2^)		
16.67 ± 5.77	26.67 ± 11.54	80.00 ± 20.00	80.00 ± 0.00	50.83	Moderately repellent (II)

^*∗*^Class of repellent effect according to the classification of McDonald et al. [46].

**Table 4:** Docking results of ligands (compounds of *R. officinalis* EO) in the targeted receptors: acetylcholinesterase (PDB: 6ARY); *Botrytis cinerea β*-tubulin (PDB: 3N2G).

Ligands	Insecticidal activity			Antifungal activity		
	6ARY receptor			3N2G receptor		
	G. score (kcal/mol)	G. e-model (kcal/mol)	G. energy (kcal/mol)	G. score (kcal/mol)	G. e-model (kcal/mol)	G. energy (kcal/mol)
1,8-Cineole	−4.665	−26.333	−20.443	−5.718	−26.651	−20.222
*α*-Caryophyllene	−6.276	−30.253	−21.981	−7.025	−29.803	−24.482
*α*-Pinene	−5.731	−20.089	−14.951	−6.007	−19.605	−12.82
*α*-Terpinene	−5.89	−26.2	−19.371	−5.75	−26.163	−19.755
*α*-Terpineol	−5.632	−29.418	−22.05	−5.497	−26.902	−21.041
*β*-Caryophyllene	−6.043	−26.567	−20.81	−6.772	−21.92	−21.162
*β*-Myrcene	−2.586	−20.545	−17.955	−2.709	−25.166	−21.865
*β*-Pinene	−5.972	−25.67	−18.99	−5.666	−14.388	−7.458
Borneol	−7.254	−34.565	−24.243	—	—	—
Bornyl acetate	−5.032	−36.539	−27.741	−6.029	−15.631	−17.282
Camphene	−5.923	−27.051	−19.988	—	—	—
Camphor	−7.023	−35.045	−24.779	−5.14	−10.395	−9.323
Limonene	−5.002	−21.261	−16.375	−4.598	−21.193	−17.769
Linalool	−3.991	−27.208	−23.225	−3.466	−27.038	−23.827
*p*-Cymene	−5.88	−27.853	−20.657	−5.512	−26.371	−19.85
Verbenone	−6.008	−31.773	−23.189	−6.213	−28.344	−21.044

## Data Availability

The data used to support the findings of the study are included within the article.
